# Adjuvant albumin-bound paclitaxel combined with S-1 vs. oxaliplatin combined with capecitabine after D2 gastrectomy in patients with stage III gastric adenocarcinoma: a phase III multicenter, open-label, randomized controlled clinical trial protocol

**DOI:** 10.1186/s12885-020-07772-7

**Published:** 2021-01-12

**Authors:** Xiangdong Cheng, Dan Wu, Nong Xu, Luchuan Chen, Zhilong Yan, Ping Chen, Lei Zhou, Jianfa Yu, Jiuwei Cui, Wei Li, Chang Wang, Wenming Feng, Yunhai Wei, Pengfei Yu, Yian Du, Jieer Ying, Zhiyuan Xu, Litao Yang, Yunli Zhang

**Affiliations:** 1 Department of Abdominal Surgery, Zhejiang Cancer Hospital (University of Chinese Academy of Sciences Cancer Hospital), Zhejiang, China; 2 Department of General Surgery, Second Affiliated Hospital of Medical College of Zhejiang University, Zhejiang, China; 3 Department of Oncology, First Affiliated Hospital of Medical College of Zhejiang University, Zhejiang, China; 4 Department of Gastrointestinal Oncology Surgery, Fujian Provincial Cancer Hospital, Fuzhou, Fujian China; 5 Department of Gastrointestinal Surgery, Ningbo First Hospital, Zhejiang, China; 6 Department of Gastrointestinal Surgery, Ningbo Second Hospital, Zhejiang, China; 7 Department of General Surgery, China-Japan Friendship Hospital, Beijing, China; 8 Department of Gastrointestinal Surgery, Zhejiang Provincial Hospital of Traditional Chinese Medicine, Zhejiang, China; 9 Oncology Central, First Hospital of Jilin University, Jilin, China; 10 Department of Hepatobiliary Pancreatic Surgery, Huzhou First People’s Hospital, Zhejiang, China; 11 Department of Hepatobiliary Pancreatic Surgery, Huzhou Central Hospital (Zhejiang University Huzhou Hospital), Zhejiang, China

**Keywords:** Gastric cancer, Adjuvant chemotherapy, Albumin-bound paclitaxel, S-1, Surgery, Survival

## Abstract

**Background:**

Surgery is the only treatment option for operable gastric cancer. The CLASSIC and ACTS-GC studies showed that the 5-year overall survival (OS) of patients with stage III gastric cancer undergoing D2 gastrectomy is still very low. Whether adjuvant nanoparticle albumin-bound paclitaxel (nab-paclitaxel) combined chemotherapy is more effective than the XELOX standard adjuvant chemotherapy in patients with stage III gastric cancer has not been confirmed.

**Methods:**

This is a multicenter, open-label, phase III clinical study. In this trial, 616 patients with locally advanced stage III gastric cancer that underwent curative D2 radical surgery and achieved R0 are planned to be included. Patients will be randomized 1:1 to nab-paclitaxel combined with S-1 (AS) vs. oxaliplatin combined with capecitabine (XELOX). XELOX group: Patients assigned to the XELOX group received eight 3-week cycles of oral capecitabine (1000 mg/m^2^) twice daily on days 1–14 of each cycle plus intravenous oxaliplatin 130 mg/m^2^ on day 1 of each cycle. AS group: AS group received eight 3-week cycles of oral S-1 (80–120 mg) (< 1.25 m^2^, 40 mg; 1.25 to < 1.5 m^2^, 50 mg; and > 1.5 m^2^, 60 mg) twice daily on days 1–14 plus intravenous nab-paclitaxel 120 mg/m^2^ on days 1 and 8 of each cycle. The primary endpoint was the 3-year disease-free survival (3-year-DFS) defined as the time from randomisation to the time of recurrence of the original gastric cancer, development of a new gastric cancer, or death from any cause. The secondary endpoints were the overall survival, (defined as the time from the date of randomisation to date of death from any cause) and safety (any adverse event).

**Discussion:**

Compared with previous studies, this study includes nab-paclitaxel based on S-1 adjuvant chemotherapy, which is expected to achieve better efficacy and lower toxicity than the standard treatment. This study is the first clinical study to evaluate the safety and efficacy of nab-paclitaxel combined with S-1 in patients with stage III gastric cancer after D2 radical resection.

**Trial registration:**

This clinical trial has been registered with ClinicalTrials.gov, registration number: NCT04135781, on October 20th, 2019.

## Background

Gastric cancer accounts for about 8.2% of cancers worldwide; about 1 million new cases were diagnosed globally in 2018, ranking fifth in global malignant tumors and ranking third in mortality [1]. More than 70% of the world’s gastric cancer occurs in developing countries, and about 50% occurs in China. In 2012, there were about 424,000 new cases of gastric cancer in China, the incidence was about 31.28/100,000, the number of deaths was about 298,000, and the mortality rate was about 22.04/100,000 [2]. China is a country with a high incidence of gastric cancer, and the burden of disease is serious. The vast majority of cases are gastric adenocarcinoma. Helicobacter pylori is a major risk factor for gastric cancer, and nearly 90% of new cases of non-cardia gastric cancers are attributed to this bacterium [3, 4].

Surgical resection is the only possible cure for patients with gastric cancer, and the prognosis of gastric cancer is closely related to the time between diagnosis and treatment. Most early gastric cancers can be treated under endoscopy, and the 5-year survival rate is more than 90%, while the 5-year survival rate of advanced gastric cancer is still less than 30% even after surgery-based comprehensive treatments [5]. Adjuvant chemotherapy after radical resection of gastric cancer can effectively prolong patient survival. In a phase III trial, 1059 patients were randomized to receive S-1 adjuvant chemotherapy or surgery alone, and the results showed that S-1 as adjuvant chemotherapy was better than surgery alone in patients with stage II/III gastric cancer after D2 lymph node dissection; the 3-year RFS rate was 72.2% in the S-1 treatment group and 59.6% in the surgery alone group [6]. In the CLASSIC study, the 3-year DFS rates in the XELOX group and the surgery alone group were 74 and 59%, respectively (HR 0.56, 95% CI 0.44–0.72; *p*< 0.0001); the survival rates were 85 and 71% in stage II (T2N1, T1N2, T3N0), 66 and 51% in stage IIIA (T3N1, T2N2, T4N0), and 61 and 33% in stage IIIB (T3N2). Therefore, more effective adjuvant chemotherapy is urgently needed in patients with stage III gastric cancer [7].

Recent studies have demonstrated the efficacy and safety of adjuvant chemotherapy based on S-1 in D2 radical surgery for postoperative adjuvant chemotherapy in patients with stage II-III gastric cancer, including S-1 plus cisplatin or S-1 plus oxaliplatin [8, 9]. A clinical trial of an AS regimen [10] in patients with advanced gastric cancer showed an ORR of 58.9%, an mPFS of 9.6 months, and an mOS of 14.6 months among 72 patients. In terms of safety, 22 patients (30.1%) developed grade 3/4 toxicity, mainly including neutropenia (12.3%), anemia (5.5%), diarrhea (6.8%), vomiting (2.7%), and peripheral neuropathy (1.4%); no fatal adverse event occurred, indicating that the AS regimen has good efficacy and controllable safety, but whether it is more effective than standard adjuvant XELOX in adjuvant chemotherapy has not been confirmed. This multicenter phase III study evaluates the safety and efficacy of nanoparticle albumin-bound paclitaxel (nab-paclitaxel) combined with S-1 in patients with stage III gastric cancer after D2 radical resection.

## Methods/design

The clinical trial is conducted in accordance with *The World Medical Association-Declaration of Helsinki* and the guidelines of Criteria for the Quality Control of Clinical Trial of Drugs promulgated by the CDE. This prospective phase III clinical trial explores the efficacy and safety of nab-paclitaxel (keaili®, CSPC Ouyi Pharmaceutical Co. Ltd.) combined with S-1 vs. oxaliplatin combined with capecitabine in the treatment of stage III gastric cancer. The trial protocol has been approved by the Review Board (IRB) of each participating institution and by the Cancer Hospital Affiliated to the Chinese Academy of Sciences. The protocol of this study has been registered in the ClinicalTrials.gov registry (NCT04135781; October 20th, 2019).

### Inclusion and exclusion criteria

Patients who were pathologically diagnosed with stage III gastric adenocarcinoma (gastroesophageal junction) after D2 radical resection and achieved R0 resection were identified. Patients who met the inclusion criteria but not the exclusion criteria were enrolled.

### Inclusion criteria

1. Aged between 18 and 75 years;
2. Patients who were histologically diagnosed with gastric adenocarcinoma (including gastroesophageal junction adenocarcinoma), stage III by pathological examination (based on the eighth edition of the AJCC Cancer Staging Manual);
3. Patients are able to provide reports of disease-related pathological diagnosis.
4. Patients underwent D2 radical resection within 6 weeks prior to random enrollment and achieved R0 resection;
5. Patients are able to receive chemotherapy within 7 days after randomization.
6. The patient had not previously received anti-tumor treatment (including systemic chemotherapy and local radiotherapy), except for surgery of the primary lesions;
7. ECOG status score 0 or 1;
8. Hematological examination shows no obvious signs of hematological disease, absolute neutrophil count (ANC) ≥1.5× 10^9^/L, platelet count ≥100× 10^9^ /L, hemoglobin (Hb) ≥90 g/L, white blood cells (WBC) ≥3.0× 10^9^/L, and no bleeding tendency before enrollment;
9. Liver function tests: alanine aminotransferase (ALT), aspartate aminotransferase (AST), and alkaline phosphatase (ALP) are all ≤2.5 × the upper limit of normal (ULN), and serum bilirubin ≤1.5 × ULN. For patients with Gilbert’s disease, serum bilirubin is ≤3 × ULN.
10. Renal function test: serum creatinine (Cr) ≤1.5 × ULN or creatinine clearance > 60 ml/min (calculated according to the Cockroft-Gault equation);
11. Patients can understand the study; patients and/or legal representatives voluntarily agree to participate in this study and signed the informed consent.

### Exclusion criteria

1. Patients who received any other study drug or participated in another clinical trial with therapeutic intention within 28 days before enrollment;
2. Patients have postoperative complications that require clinical intervention and affect treatment, such as gastroparesis, dumping syndrome, etc.;
3. Patients known to be allergic or intolerant to the study drugs;
4. Uncontrolled serious medical conditions that the investigator believes will affect the subject’s treatment regimen, such as a combination of serious medical conditions, including severe heart disease (New York Heart Association (NYHA) level II or more severe congestive heart failure), cerebrovascular disease, uncontrolled diabetes, uncontrolled high blood pressure, uncontrolled infection, etc.;
5. Known history of HIV;
6. Known active HCV and HBV infection.
7. Patients had a malignant tumor other than gastric cancer in the past 5 years (except for the current gastric cancer); if it meets all the following criteria, it is qualified: treatment of malignant tumors for cure, such as fully treated cervical carcinoma in situ, non-melanoma skin cancer, or localized prostate cancer after radical operation (PSA ≤10 ng/ml); and no signs of recurrence or metastasis were found according to the imaging results of follow-up and any disease-specific tumor markers;
8. Patients accompanied by dysphagia, complete or incomplete gastrointestinal obstruction, active gastrointestinal bleeding, or perforation;
9. Female patients during pregnancy or lactation, or subjects of childbearing age who refuse contraception;
10. Patients who were not suitable for the enrollment of this study judged by the investigator.

### Randomization

Eligible patients will be randomly assigned to the AS or XELOX group at a ratio of 1:1. The AS group will receive nab-paclitaxel plus S-1, and the XELOX group will receive oxaliplatin plus capecitabine (Table 1). Randomization will be performed using an interactive speech/network response system, and patients will be stratified by histological differentiation type (differentiated vs. undifferentiated) and tumor stage (IIIA vs. IIIB, IIIC). This study is an open study.

**Table 1:** Therapeutic regimen

Regimen	Dose	Course of treatment	Treatment modalities	Administration time
AS group				
Paclitaxel (albumin-bound)	120 mg/m^2^	Q3W	IV	D1, 8
S-1	< 1.25 m^2^, 40 mg; 1.25m^2^ to < 1.5 m^2^, 50 mg; and > 1.5 m^2^, 60 mg	Q3W	Oral	D1–14
XELOX group				
Oxaliplatin	130 mg/m^2^	Q3W	IV	D1
Capecitabine	1000 mg/m^2^	Q3W	Oral	D1–14

### Intervention

**Fig. 1:**
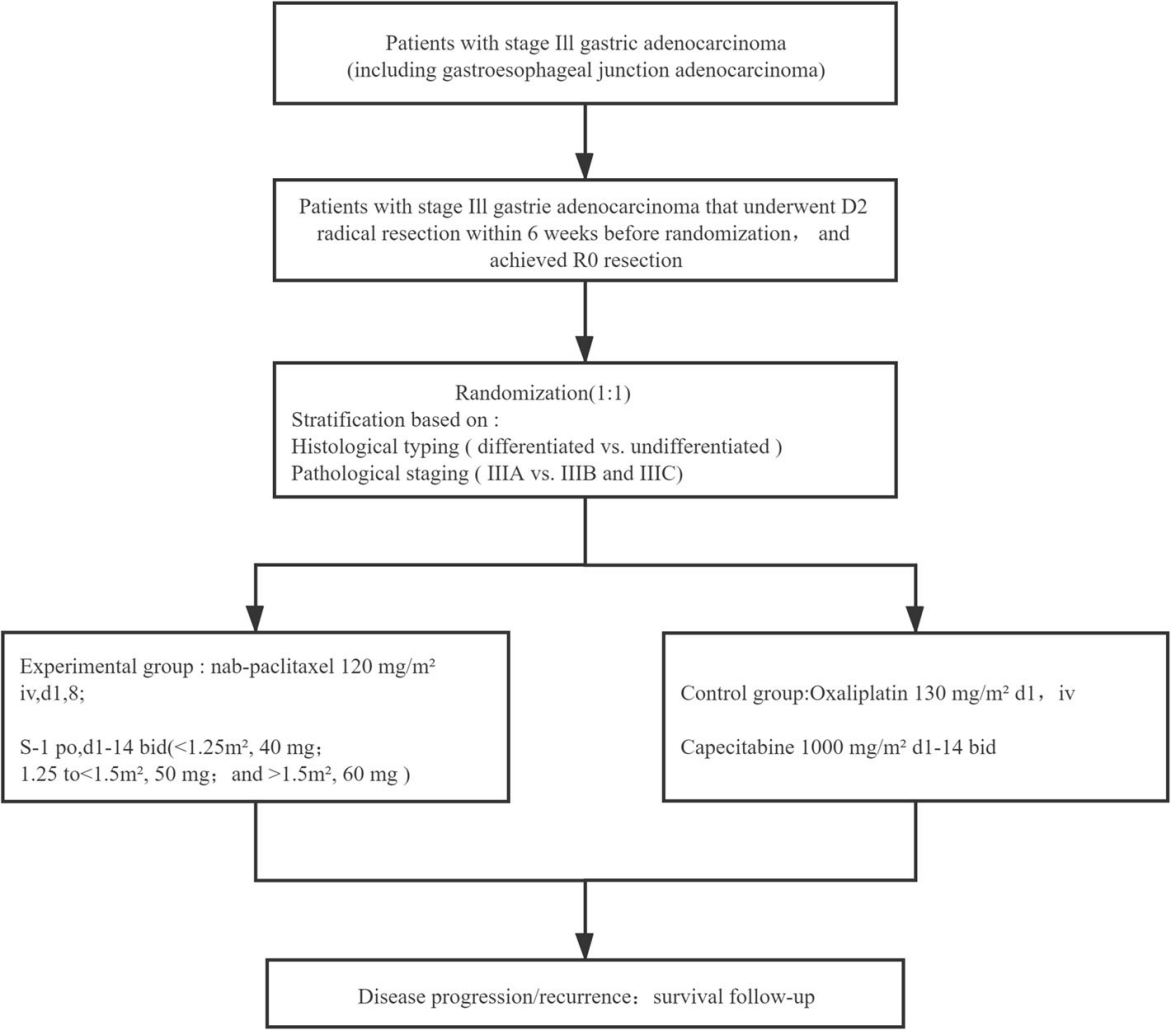
Study flowchart

This multicenter, open-label, randomized controlled phase III clinical study (Fig. 1) evaluates the efficacy and safety of nab-paclitaxel combined with S-1 (AS) vs. oxaliplatin combined with capecitabine (XELOX) in patients with stage III gastric cancer who underwent D2 radical surgery and achieved R0 resection. In the XELOX group, capecitabine (1000 mg/m^2^) will be administered orally twice daily on days 1–14, with oxaliplatin given i.v. at 130 mg/m^2^ on day 1 of each 3-week cycle for eight cycles. In the AS group: S-1 (80–120 mg) (< 1.25 m^2^, 40 mg; 1.25 to < 1.5 m^2^, 50 mg; and > 1.5 m^2^, 60 mg) will be was administered orally twice daily on days 1–14, with paclitaxel (albumin-binding) given i.v. at 120 mg/m^2^ on days 1 and 8 of each 3-week cycle for eight cycles.

### Study endpoints

The primary endpoint is DFS. The secondary endpoints are OS and safety (occurrence of adverse events per treatment cycle).

### Follow-up

All patients will be followed according to the protocol (i.e., once every 3 months). Physical examination, tumor marker examination, and chest X-ray examination will be performed every 3 months. Computed tomography (CT) or magnetic resonance imaging (MRI) scans will be performed every 3 months for the first 2 years and every 6 months for the 3rd year. Endoscopy will be performed once a year.

### Sample size

In this trial, the patients will be hierarchically grouped by a network multicenter central stochastic system. The subjects will be randomized by the system after screening. This study uses DFS as the main evaluation index. According to the CLASSIC study, the 3-year DFS rates of patients with IIIA and IIIB gastric adenocarcinoma who received XELOX regimen are 66 and 61%, respectively [7]. According to the ACTS-GC trial, the 3-year recurrence-free survival (RFS) rate of patients with stage III gastric cancer who received S-1 adjuvant chemotherapy (sixth edition of International Union Against Cancer (UICC) TNM classification) is 69.1% in stage IIIA and 44.8% in stage IIIB [11]. The 3 year-DFS rate of patients with stage III gastric adenocarcinoma who received nab-paclitaxel combined with S-1 treatment is estimated to be 71%. Considering type I error (bilateral) α=0.05 and the test efficacy β=0.8, hazard ratio (HR) of 0.66, and estimated drop-out rate of 10%, about 616 patients will be randomly enrolled in the clinical study, with a total study period of about 60 months.

### Statistical analysis

The efficacy analysis will be performed in the intention-to-treat (ITT) population. The DFS will be calculated from the randomization date to the first detected disease recurrence date. The following events are defined as recurrence: primary cancer recurrence, newly diagnosed gastric cancer, and death. The OS will be calculated from the randomization date to the date of death or the date of last follow-up. Survival rates will be estimated using the Kaplan-Meier method, and the differences between survival curves will be tested using the log-rank test. An accurate 95% confidence interval of the overall survival and safety analysis will be estimated. All statistical tests will be two-sided tests. A significant difference will be considered when *P*< 0.05. The risk ratio will be estimated using a stratified Cox regression model. In this study, 616 patients are expected to be enrolled. An interim analysis is planned when the number of events will reach 117. This clinical trial will be terminated under the condition that the 95% confidence interval (CI) of HR spans 1 and P>α=0.0084. The overall test level of this study is α=0.05 (two-sided) according to the O’Brien Flemming method, and α distribution is performed based on the number of endpoint events collected during the interim analysis. The mid-term analysis α will be 0.0084, and the significant level of the final analysis will be α=0.0468. The results of efficacy and safety will be reviewed by the external data monitoring committee to determine whether the study should continue, according to predetermined criteria.

## Discussion

The aim of this study is to compare the effect of adjuvant chemotherapy with nab-paclitaxel plus S-1 versus oxaliplatin plus capecitabine for gastric cancer after D2 gastrectomy, so as to explore which treatment is better. Although this is not the first phase III trial comparing postoperative adjuvant chemotherapy worldwide, it has some advantages. XELOX regimen improves patient outcomes after surgery and is the preferred adjuvant chemotherapy after curative resection of stage III gastric cancer recommended by the Chinese Society of Clinical Oncology (CSCO) guidelines [12]. However, most patients can not complete the anticipated treatment due to therapeutic toxicity. S-1 monotherapy has proven to be effective and low-toxic, and is preferred in some Asian countries as standard component of resectable gastric cancer therapy [6]. Nab-paclitaxel is confirmed effective and low-toxic, and is approved for the treatment of advanced gastric cancer [10]. Therefore, we choose XELOX as the control group and hypothesized that the combination of nab-paclitaxel and S-1 could improve the efficacy of adjuvant chemotherapy compare with XELOX and has better safety.

We keep following with global trend. Result from JCOG 1104 [13], which is randomized trial comparing four and eight courses of adjuvant S-1, confirmed that 1-year’s S-1 treatment should be the standard adjuvant chemotherapy for stage II gastric cancer. The trial also suggested S-1 monotherapy still needs to be further optimized. The ARTIST2 trial [14] include patients with stage II and III gastric cancer, suggesting that SOX is superior to S-1 monotherapy, but the subgroup data still not be avaliable and the outcomes for patients with stage III is unknown. JACCRO GC07 trial [15] showed that the addition of docetaxel to S-1 is effective in patients with stage III gastric cancer. Compared with S-1 monotherapy, the 3-year RFS improved from 50 to 66%, Although these studies have proved XELOX may not be the optimal regimen in other countries, but in China we can not fully accept considering different patients characteristic, so the further investigation is necessary. We conduct this trial aimed at in patients with stage III disease, expect to expand and optimize the existing therapies. And the trial of comparison with docetaxel combined S-1 and SOX may also be carried out in combination with our research process.

Taken all together, the results of this study will provide prospective multicenter data for patients with stage III gastric cancer in China and can be recommended as a standard component of postoperative adjuvant chemotherapy. It will help establish postoperative clinical outcomes for patients with locally advanced gastric adenocarcinoma or gastroesophageal junction adenocarcinoma.

## Data Availability

The datasets used and/or analysed during the current study are available from the corresponding author on reasonable request.

## Abbreviations

AS: Nab-paclitaxel and S-1
BSA: Body surface area
CT: Computed tomography
DFS: Disease-free survival
OS: Overall survival
RFS: Recurrence-free survival
XELOX: Capecitabine and oxaliplatin
